# Use of Grape Pomace Phenolics to Counteract Endogenous and Exogenous Formation of Advanced Glycation End-Products

**DOI:** 10.3390/nu11081917

**Published:** 2019-08-15

**Authors:** Pedapati S. C. Sri Harsha, Vera Lavelli

**Affiliations:** 1 Institute of Food and Health, University College Dublin, Dublin, Dublin 4, Ireland; 2 DeFENS, Department of Food, Environmental and Nutritional Sciences, Università degli Studi di Milano, via Celoria 2, 20133 Milano, Italy

**Keywords:** advanced glycation end-products, Maillard reaction, N*ε*-(carboxymethyl) lysine, grape phenolics

## Abstract

The increase in consumption of “ultra-processed” foods has raised attention because of the possible adverse effects deriving from the Maillard reaction leading to the formation of toxic advanced glycation end-products (AGEs) during food processing. Additionally, the increasing trend and consumption of sugar-added foods and sweetened beverages is related to the endogenous formation of the same toxic compounds. However, ultra-processing in the context of food technology can bring challenges as well as a wealth of opportunities. Indeed, re-processing of grape pomace, a by-product of winemaking, can yield phenolic-rich fractions that efficiently counteract the effects of AGEs. In this review, the process of endogenous and exogenous AGE formation is illustrated. Then, the ability of grape phenolics to act as inhibitors of AGE formation is presented, including the efficacy ranking of various individual compounds measured in vitro and the outcome of in vivo double-blinded randomized crossover trials designed to prove the efficacy of grape phenolics as inhibitors of protein carbonylation. Finally, a survey of model functional foods added with grape phenolics, either to lower the dietary load of AGEs or to deliver antiglycation agents in vivo is listed in order to highlight the opportunity to develop safe and tailor-made “anti-AGEs” food applications.

## 1. Endogenous Formation of Advanced Glycation End-Products (AGEs)

The process of non-enzymatic protein glycation plays a major role in a number of pathological processes [1,2,3,4,5,6,7]. Protein glycation occurs slowly and continuously throughout the life span, causing the accumulation of advanced glycation end-products (AGEs) in elderly people, which has been involved in the pathogenesis of age-related diseases. Apart from elderly people, in diabetic people, insulin resistance and hyperglycaemia accelerate the accumulation of AGEs, leading to early development of comorbidities [1,2]. AGEs have also been found to be involved in cardiovascular diseases [2,3], chronic renal diseases [4], chronic liver diseases and hepatocellular carcinoma [7].

**Figure 1:**
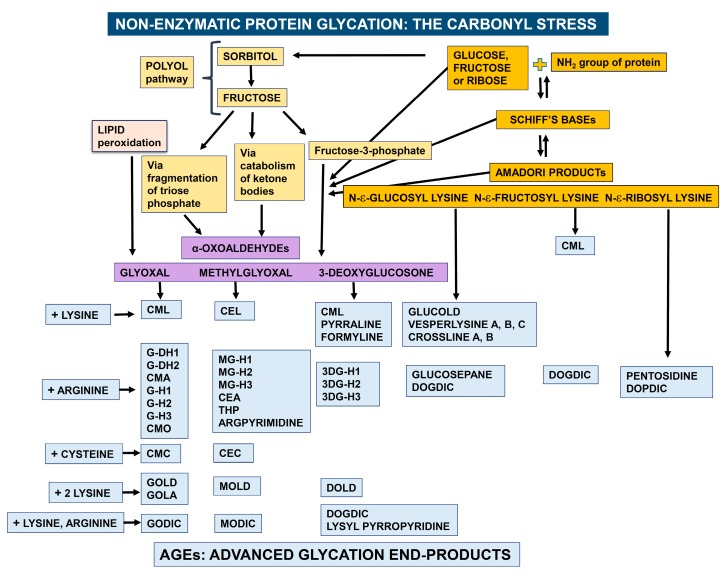
Schematic representation of non-enzymatic protein glycation. Details of these degradation patterns have been reported elsewhere [7,8]. Legend for AGE abbreviations is reported in Box 1.

The pattern of protein glycation in vivo is complex. This process is initiated by the reaction of a carbonyl group from a reducing sugar, such as glucose, fructose or ribose, with free amino groups of proteins to form Schiff bases, which then undergo an Amadori rearrangement. The resulting Amadori products degrade further to form reactive α-dicarbonyls such as methylglyoxal, glyoxal and 3-deoxyglucosone. These harmful compounds can also be generated through other routes such as the polyol pathway and lipid peroxidation. Then, α-dicarbonyls react with one single amino group of the side chains of lysine and arginine or with the thiol group of cysteine in proteins or, alternatively, cross-link two of these residues. These reactions lead to the formation of various cross-linked adducts, which are collectively called AGEs. The Amadori products can also rearrange directly to form AGEs (Figure 1) [7,8].

Box 1 **Nomenclature for the most common AGEs.**

**GLYOXAL + LYSINE:**

**CML**: Nε-(carboxymethyl) lysine

**GLYOXAL + ARGININE:**

**G-DH1**: Nẟ-(3,4-dihydroxy-1-imidazolidin-2-yl) ornithine
**G-DH2**: 5-(4,5-dihydroxy-2-imino-1-imidazolidinyl) norvaline
**CMA**: Nω-(carboxymethyl) arginine
**G-H1**: Nẟ-(5-hydro-4-imidazolon-2-yl) ornithine
**G-H2**: 5-(2-amino-5-hydro-4-imidazolon-1-yl) norvaline
**G-H3**: 5-(2-amino-4-hydro-5-imidazolon-1-yl) norvaline
**CMO**: Nẟ-(carboxymethyl) ornithine

**GLYOXAL + CYSTEINE:**

**CMC**: S-(carboxymethyl) cysteine

**GLYOXAL + TWO LYSINE RESIDUES:**

**GOLD**: glyoxal-lysine dimer, 6-{1-[(5S)-5-ammonio-6-oxido-6-oxohexyl]imidazolium-3-yl}-L-norleucine
**GOLA**: Nε-(2-{[(5S)-5-ammonio-6-oxido-6-oxohexyl] amino}-2-oxoethyl)-L-lysine

**GLYOXAL + ARGININE AND LYSINE:**

**GODIC**: Nε-(2-{[(4S)-4-ammonio-5-oxido-5-oxopentyl]amino}-3,5-dihydro-4H-imidazol-4-ylidene)-L-lysine

**METHYLGLYOXAL + LYSINE:**

**CEL**: Nε-(carboxylethyl) lysine

**METHYLGLYOXAL + ARGININE:**

**MG-H1**: Nẟ-(5-methyl-4-imidazolon-2-yl)-L ornithine
**MG-H2**: 2-amino-5-(2-amino-5-hydro-5-methyl-4-imidazolon-1-yl)pentanoic acid
**MG-H3**: 2-amino-5-(2-amino-4-hydro-4-methyl-5-imidazolon-1-yl)pentanoic acid
**CEA**: Nω-(carboxyethyl) arginine
**THP**: Nẟ-(4-carboxy-4,6-dimethyl-5,6-dihydroxy-1,4,5,6-tetrahydropyrimidine-2-yl)-L-ornithine
**ARGPYRIMIDINE**: Nẟ-(5-hydroxy-4,6-dimethylpyrimidine-2-yl)-L-ornithine

**METHYLGLYOXAL + CYSTEINE:**

**CEC**: S-carboxyethylcysteine

**METHYLGLYOXAL + TWO LYSINE RESIDUES:**

**MOLD**: methylglyoxal lysine dimer, 6-{1-[(5S)-5-ammonio-6-oxido-6-oxohexyl]-4-methyl-imidazolium-3-yl}-L-norleucine

**METHYLGLYOXAL + LYSINE AND ARGININE:**

**MODIC**: 2-ammonio-6-({2–[4-ammonio-5-oxido-5-oxopently)amino]-4-methyl-4,5-dihydro-1H-imidazol-5-ylidene}amino)hexanoate

**3-DEOXYGLUCOSONE + LYSINE:**

**CML**: Nε-(carboxymethyl) lysine
**PYRRALINE**: 6-(2-formyl-5-hydroxymethyl-1-pyrrolyl)-L-norleucine
**FORMYLINE**: 6-(2-formyl-1-pyrrolyl)-L-norleucine

**3-DEOXYGLUCOSONE + ARGININE:**

**3DG-H1**: Nẟ-[5-hydro-5-(2,3,4-trihydroxybutyl)-4-imidazolon-2-yl] ornithine
**3DG-H2**: 5-[2-amino-5-hydro-5-(2,3,4-trihydroxybutyl)-4-imidazolon-1-yl] norvaline
**3DG-H3**: 5-[2-amino-4-hydro-4-(2,3,4-trihydroxybutyl)-5-imidazolon-1-yl] norvaline

**3-DEOXYGLUCOSONE + TWO LYSINE RESIDUES:**

**DOLD**: 1,3-di(Nε-lysino)-4-(2,3,4-trihydroxybutyl)-imidazolium

**3-DEOXYGLUCOSONE + ARGININE AND LYSINE:**

**DOGDIC**: Nε-{2-{[(4S)-4-ammonio-5-oxido-5-oxopentyl]amino}-5-[(2S,3R)-2,3,4-trihydroxybutyl]-3,5-dihydro-4H-imidazol-4-ylidene}-L-lysinate
**LYSYL-PYRROPYRIDINE**: lysyl-3,3a,8,8a-tetrahydro-3a-hydroxy-2-(1,2-dihydroxyethyl)-5 hydroxymethyl-2*H*-furo [3′,2: 4,5]pyrrolo-[2,3-*c*]-pyridinium

**DEGRADATION OF AMADORI PRODUCTS**

**GLUCOSEPANE**: 2-acetylamino-5-[(4-butyl-6,7-dihydroxy-4,5,6,7,8,8ahexahydroimidazo [4,5-b]azepin-2-yl)amino]-pentanoic acid
**PENTOSIDINE**: 6-[2-[[(4S)-4-amino-5-hydroxy-5-oxopentyl] amino]-4-imidazo [4,5-b]pyridinyl]-L-norleucine
**GLUCOLD**: 1,3-bis-(5-amino-5-carboxypentyl)-4-(1,2,3,4-tetrahydroxybutyl)-3H-imidazolium
**CROSSLINES A and B** N-diacetates: (3R,4S)-3,4-dihydroxy-5-[(1S or 1R,2S,3R)-1,2,3,4-tetrahydroxybutyl]-1,7-bis[6-(N-acetyl-L-norleucyl)]-1,2,3,4-tetrahydro-1,7-naphthyridinium chloride
**DOPDIC**: Nε-{2-{[(4S)-4-ammonio-5-oxido-5-oxopentyl] amino}-5-[(2S)-2,3-dihydroxypropyl] 3,5-dihydro-4H-imidazol-4-ylidene}-L-lysinate
**VESPERLYSINE A**: 6-hydroxy-1,4-di{6-(L-norleucyl)}-1H-pyrrolo[3,2-b] pyridinium
**VESPERLYSINE B**: 6-hydroxy-5-methyl-1,4-di{6-(L-norleucyl)}-1H-pyrrolo[3,2-b] pyridinium
**VESPERLYSINE C**: 5-hydroxymethyl-1,6-di{6-(L-norleucyl)}-1H-pyrrolo[3,4-b] pyridinium

AGEs are bound to proteins with high contents of lysine and arginine units and long half-lives such as collagen, serum albumin, elastin, myelin, low density lipoprotein, plasminogen activator, fibrinogen, etc. These glycated proteins lose their functionality and are resistant to removal by proteolytic enzymes. Accumulation of AGEs in the extracellular matrix causes abnormal cross-linking and results in a decrease of elasticity in vessels leading to arterial stiffness.

In addition, AGE receptors (RAGE) are expressed on many cell types. AGE–RAGE interaction leads to the generation of reactive oxygen species, inflammation, angiogenesis and proliferation through various signal transduction pathways. The activation of RAGE stimulates the phosphorylation of the extracellular signal-regulated kinases, finally resulting in the stimulation of transcription factor NF-κB and the production of pro-inflammatory cytokines. In addition, the stimulation of RAGE results in the activation of the transforming growth factor β (TGF-β) pathway and induces vascular endothelial growth factor overexpression. Intracellular AGE formation can also lead to quenching of nitric oxide and impaired function of growth factors [9].

In contrast, other AGE receptors (AGE-Rs) form a complex of distinct receptors, which are responsible for the detoxification and clearance of AGEs [10,11,12]. A fundamental role in the AGE detoxification system is also played by the enzymatic glyoxalase system, which comprises glyoxalase-I and glyoxalase-II. Glyoxalase-I catalyses the conversion of α-oxo-aldehydes such as methylglyoxal into the corresponding hemithioacethal S-D lactoylglutathion, using L-glutathione (GSH) as a cofactor. Then, glyoxalase-II hydrolyses the reaction of S-D-lactoylglutathion to H_2_O and d-lactate, with the regeneration of GSH [5]. Hence, both the glyoxalase inducers and competitive inhibitors, which modulate the functionality of these systems, are of interest for the therapy of AGE-related diseases [13].

## 2. Effect of Diet on the Formation of AGEs

Protein glycation known as the Maillard reaction also occurs in heat-treated foods, generating AGEs that are then exogenously introduced with the diet [14]. AGEs are naturally present in uncooked animal-derived foods, and cooking results in the formation of new AGEs within these foods. Nε-(carboxymethyl) lysine (CML) level was found to be a useful marker of dietary AGE content and chosen to characterize the AGE load of foods [7]. Investigation on CML content of 549 foods demonstrated that grilling, broiling, roasting, searing and frying accelerate new AGE formation [15]. These modified proteins are resistant to digestion but can be fermented by the colonic bacteria. Upon fermentation, AGEs form toxic compounds that promote the beginning or maintenance of ulcerative colitis in the host. This latter inflammatory disease causes mucosal necrosis [16].

In vivo studies show that dietary AGEs directly correlate with circulating AGEs, such as CML, as well as with markers of oxidative stress [17]. Accordingly, restriction of dietary AGEs in patients with diabetes [18] reduces markers of oxidative stress and inflammation. Moreover, obese individuals with metabolic syndrome were reported to have elevated serum AGEs that strongly correlate with insulin resistance, oxidative stress and inflammation [19]. Interestingly, a diet low in AGEs does not cause a major reduction in adiposity but improves insulin resistance in obese people with metabolic syndrome and may decrease the risk of type 2 diabetes [18].

In another hypothesis, protein glycation also occurs in the intestine when foods and beverages sweetened with high fructose corn syrup are consumed [20]. Then, in situ enteral formed AGEs are absorbed and may contribute to inflammatory diseases via engagement of the pro-inflammatory RAGEs. Animal models of high sugar consumption, in particular fructose, have reported AGE accumulation in different tissues in association with peripheral insulin resistance and lipid metabolism alterations. Thus, among the sugars mostly used for sweetening of foods and drinks, fructose might represent the most hazardous one for AGE accumulation. Recent cross-sectional studies demonstrated that the consumption of high fructose corn syrup or fructose-sweetened beverages is associated with asthma and bronchitis in adults and with asthma in children [21,22].

**Figure 2:**
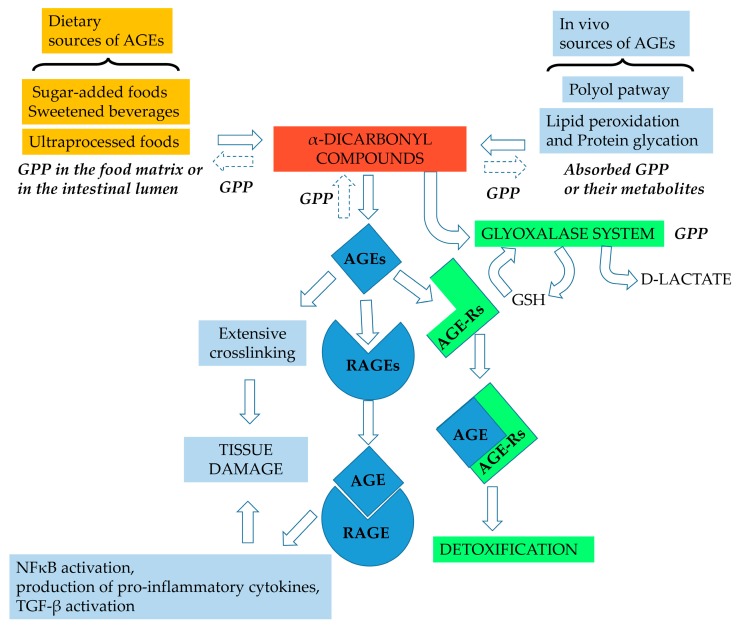
Potential sites of action of grape pomace phenolics (GPP) in the glycation pathway. In the food matrix during processing and in the intestinal lumen, GPP can inhibit α-dicarbonyl compounds and AGE formation by acting as metal chelators, antioxidants and carbonyl trapping agents. Absorbed GPP or their metabolites can exert the same activities in vivo; moreover, GPP can assist the detoxifying process in vivo by acting as a glyoxalase inducer. GSH, reduced glutathione; NFκB, nuclear factor kappa-light-chain-enhancer of activated B cells, TGF-β transforming growth factor β.

A schematic view of the glycation process is shown in Figure 2, which also highlights the possible roles played by grape pomace phenolics, which are discussed in the following paragraphs.

## 3. Detection of AGEs

The methods to detect AGEs in human tissues, biological fluids and foods are mostly based on fluorescence spectroscopy, chromatography, or immunoassay-based procedures. Fluorescence spectroscopy can only detect fluorescent AGEs, among which the most studied are pentosidine, crosslines A and B, argpyrimidine, vesperlysines A, B, and C, and lysyl-pyrropyridine. The excitation wavelength for AGEs is generally between 300 nm and 420 nm and the emission wavelength is between 350 nm and 600 nm [23,24].

For chromatographic methods, the sample needs to be hydrolysed because most AGEs are not free but bound to proteins. To detect bound AGEs, acid hydrolysis or enzymatic hydrolysis has been applied. For the first approach, samples are first reduced in NaBH_4_ solution to prevent fructoselysine from converting to CML; trichloroacetic acid or chloroform-methanol (2:1, v:v) is then used to precipitate proteins before hydrolysis in 6 M HCl solution at 110 °C, followed by solid-phase extraction to purify the hydrolysate. Enzymatic hydrolysis prevents destruction of AGEs (such as pyrraline) that are not stable under acidic conditions. The enzymes used are pepsin, pronase E, prolinase and aminopeptidase M, respectively [23]. Then, gas chromatography-mass spectrometry (GC-MS) and high-performance liquid chromatography (HPLC) or ultra-performance liquid chromatography (UPLC) coupled with diode array detector (DAD), fluorescence detector (FLD), mass spectrometry (MS) or tandem mass spectrometry (MS/MS) are applied for the identification of the hydrolysed compounds. A FLD provides higher sensitivity that a DAD. However, while fluorescent AGEs can be detected directly, non-fluorescent AGEs require derivatization [25]. Alternatively, MS and tandem MS detectors do not require derivatization and are highly sensitive [26].

AGEs are immunogenic and hence another approach for their detection is by immunochemical methods. Both polyclonal and monoclonal antibodies to AGEs are commercially available. Among monoclonal antibodies, anti-pentosidine, anti-3-DG-H, anti-CML, anti-CEL, anti-pyrraline, anti-CMA and anti-human RAGE are commercially available [24].

## 4. Grape Pomace Phenolics as Inhibitors of Protein Glycation—Mechanisms of Action

**Figure 3:**
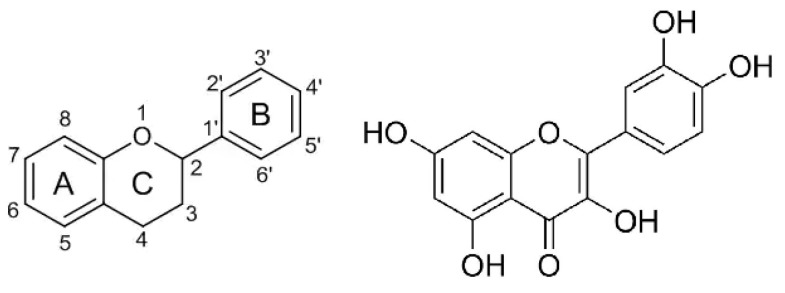
Common structure of flavonoids consisting of C(6)–C(3)–C(6) skeleton containing two aromatic rings (A,B) and one heterocyclic ring (C) with an oxygen atom (on the left) and the structure of quercetin (on the right), a member of the flavonoid family possessing the best structural requirements for α-dicarbonyl trapping ability (i.e., the hydroxyl group at C-5 on the A ring and the double bond between C-2 and C-3 on the C ring) [31].

Phenolic compounds are found to inhibit protein glycation. The inhibitory effect could be related to their metal chelation activity, since the Maillard reaction is promoted by metal ions [27]. The inhibitory mechanism of phenolics against glycation is also due to their antioxidant properties. In fact, protein glycation is accompanied by oxidative reactions, whose occurrence was proven by using phenyl-tert-butyl-nitron as a spin-trapping agent and monitoring the time course of the reaction by electron spin resonance [28]. Moreover, phenolics also act as carbonyl trapping agents [29]. For flavonoids, the structural requirements for maximum anti-glycation activity, are not the same as for antioxidant activity. Indeed, the antiglycation activity of phenolic-rich extracts from various plants is not correlated to their antioxidant activity [30]. The structure–activity relationship of flavonoids in scavenging reactive methylglyoxal was thoroughly investigated using model flavonoid molecules [31]. This latter study evidenced that: (1) the A ring is the active site of flavonoids in contributing to the methylglyoxal trapping efficacy, and the hydroxyl group at C-5 on the A ring enhances the trapping efficacy; (2) the double bond between C-2 and C-3 on the C ring could facilitate the trapping efficacy; and (3) the number of hydroxyl groups on the B ring does not significantly influence the trapping efficacy (Figure 3). Additionally, based on LC-MS studies, it was hypothesized that methylglyoxal conjugation should occur at positions C-6 and C-8 of the A ring [31,32].

Grape pomace, which is the main by-product of winemaking constituted by skins and seed, is a rich source of flavonoids. The current knowledge on the possible antiglycation activity of grape pomace phenolics was investigated by analysing the literature of the last 10 years indexed by the Scopus and Web of Science databases, using the search term “glycation” in combination with the terms “grape”, or “proanthocyanidin”, “anthocyanin”, “quercetin”, “epicatechin”, “catechin”, “resveratrol”. Grape pomace phenolics are mainly comprised of the polymeric flavonoids proanthocyanidins and the monomeric flavanols, anthocyanins and flavonols and, in minor amounts, phenolic acids and stilbenes. Methylglyoxal trapping ability was demonstrated for both red and white grape skin extracts and found to be higher in red grape skin when compared with white grape skins. For red grape skin extracts of various grape varieties, a significant correlation was found among the carbonyl trapping ability, total phenolic content and antioxidant capacity. However, for white grape skin extract from various grape varieties no correlations were observed, which suggests that other compounds were involved in the carbonyl trapping activity and/or synergism among phenolic compounds [33]. In fact, the study of model flavonoid molecules showed that they exert additive methyl glyoxal-trapping effects [31]. Hence, evaluation of the effectiveness of a complex pool of phenolics can reveal the most promising food sources.

The ability of grape skin phenolics to protect proteins from glycation was demonstrated by a model system composed of bovine serum albumin (BSA) as a protein target and fructose or methylglyoxal as glycating agents. In fact, isoelectric focusing proved that grape phenolics are able to protect the target protein from charge variation that occurs at the beginning of the glycation, due to the reaction of amino groups with fructose. Sodium dodecyl sulfate (SDS)-electrophoresis also provided evidence of the effectiveness of grape phenolics in inhibiting structural damage of BSA, since they prevent the formation of crosslinking among proteins that occurs at a later stage of the glycation reaction [34]. Fluorescence was evaluated to monitor the time course of the glycation reaction, revealing that the phenolic extracts from red and white grape skins have higher anti-glycation activity than those of commercial nutraceutical preparations. By using standard compounds, the following efficacy ranking was observed for anti-glycation activity: quercetin 3-O glucoside > malvidin 3-O glucoside > catechin > procyanidin A2. Interestingly, all grape skin phenolics were much more efficient than the synthetic inhibitor aminoguanidine. Cinnamon, sage and rosemary, were also found to be efficient antiglycation agents, but grape skin phenolics are more cost-effective [35].

## 5. Grape Pomace Phenolics as Inhibitors of Protein Glycation—In Vivo Studies

The effects of grape phenolics as inhibitors of protein carbonylation was investigated in seven in vivo randomized crossover trials (Table 1). In the first study, 15 healthy subjects received a red grape skin extract powder that delivered 31 mg/day of total phenolic compounds, for two weeks. Following grape skin extract intake, there was an increase in activities of some enzymes involved in antioxidant defense, namely, the glutathione reductase and peroxidase. No effects were observed on 2-aminoadipic semialdehyde, a plasma protein oxidation product as well as on malonyldialdehyde, a marker of lipoprotein oxidation [36], but the amount of grape skin polyphenols assumed daily was low compared to that used in later studies.

In fact, in a successive intervention study, 32 type-2 diabetes mellitus patients received a grape seed extract delivering 0.6 g⁄day of total phenolics or placebo for four weeks. Following grape seed extract intake (but not placebo), there was a significant improvement in the markers of inflammation and glycaemia and oxidative stress such as total antioxidant status and fructosamine [37].

In contrast, a lower amount of grape polyphenols was supplemented to 75 patients with stable coronary artery disease for a one-year study. In this approach, the aim was to investigate specifically the role of resveratrol, hence one group of patients was given 66.8 mg/day of grape phenolics without resveratrol and another group was given the same amount of grape phenolics along with 8.1 mg/g of resveratrol, while a third group (control) was given neither grape phenolics nor resveratrol. In subjects who received resveratrol and other grape phenolics, the anti-inflammatory protein serum adiponectin increased, plasminogen activator inhibitor type 1 increased and atherothrombotic signals in peripheral blood mononuclear cells were inhibited. The presence of resveratrol among grape phenolics appeared essential to exert these effects. However, no effect was observed on glycated haemoglobin content [38], which could be due to the low amount of polyphenols given to the patients [38].

In a later study, 38 first-degree relatives of type-2 diabetic patients were supplemented with a high-fructose diet along with 2 g/day of grape polyphenols in microcrystalline cellulose capsules or a placebo for nine weeks. Results from this study demonstrated that grape phenolics protect against fructose-induced oxidative stress, protein carbonylation and lipid peroxidation, and help prevent insulin resistance [39].

Then, a study was performed on 38 subjects with at least one diagnostic criterion of metabolic syndrome. The intervention group received 20 g/day of red wine grape pomace as a food supplement, consisting of 10 g of dietary fibre, and 0.8 g of polyphenols. Both groups maintained their regular eating habits and lifestyles. The inclusion of grape pomace in a regular diet improved blood pressure, glycaemia and postprandial insulin and decreased oxidative protein damage [40].

In another approach, 29 overweight and obese subjects were involved in an intervention study with capsules of trans-resveratrol (90 mg/day), which is found in grapes and hesperidin (120 mg/day), which is present in oranges or placebo capsules for eight weeks. Before this clinical trial, the combination of these two bioactive compounds was optimized in a cell study based on their ability as glyoxalase-1 inducers. Results from the clinical trial confirmed that the combination of both bioactive compounds increased expression and activity of glyoxalase-1, decreased plasma methylglyoxal and total body methylglyoxal-protein glycation, and decreased fasting and postprandial plasma glucose, while it increased insulin sensitivity and improved markers for arterial health. However, in previous clinical evaluations, trans-resveratrol and hesperidin used individually were ineffective [41].

Finally, 37 apparently healthy, non-smoking adults were involved in an intervention study with two bioactive compounds that are quite abundant in grapes (i.e., (−)-epicatechin (100 mg/day), quercetin 3-glucoside (160 mg/day)) or placebo capsules for periods of four weeks. This study demonstrated that quercetin, but not epicatechin, decreased plasma methylglyoxal concentrations. Quercetin may potentially form a new treatment strategy for diseases in which methylglyoxal is involved [42]. All together these studies demonstrate that grape phenolics can play a role in the defense system against protein glycation, although further information on the synergism among these bioactive molecules is needed to provide mechanistic insights on the role of grape phenolics.

## 6. Food Formulation with Grape Pomace Phenolics as a Strategy to Counteract AGE Formation

Food formulation strategies with grape pomace phenolics have been proposed to counteract AGEs formation in foods. Bakery products are among the most studied foods in which AGEs can be generated through intensive heat treatment. CML level was found to be 35 mg/kg in bread crust [43]. In another study, CML level was found to be 49.72 mg/kg in bread crust and 15.09 mg/kg in bread crumbs [44]. In addition to the intensity of heat treatment, the ingredients used play an important role in CML formation, as evidenced from a study in which a basic muffin recipe was modified to assess the effect of individual ingredients on AGE formation [30]. The basic mixture of wheat flour, water, sugar and fat in the ratio usually used for preparing muffins generated a CML level of 26.55 mg/kg muffins. The sugar type has a fundamental importance in AGE formation: the muffins made with glucose had the highest levels and approximately 3.5-fold greater content than those with fructose; the muffins made with raw cane sugar produced about 11.5-fold higher concentrations of CML than the white beet sugar-formulated muffins, probably due to higher levels of metal ions than white beet sugar, which promote the Maillard reaction. The degree of unsaturation of the oils also influenced the total amount of CML formed. In fact, the highest content of CML was detected in the muffins made with grapeseed oil, while the lowest content of CML was found in samples made with olive oil [30].

In view of grape phenolic efficacy as an antiglycation agent, some studies have proposed the use of grape phenolics in food matrices in order to decrease process-induced AGE formation and hence, AGE load with foods (Table 2). In one study, 2 g/kg grape seed extract powder was used in bread to prevent AGE formation during cooking. This incorporation of grape seed extract led to a favourable change in the colour of bread without causing significant changes in other sensory properties, and decreased CML level by 50% [43]. In following studies, the addition level of phenolics in bakery products was increased by more than 10-fold. Purified phenolics, namely, quercetin, naringenin, epicatechin, rosmarinic acid and chlorogenic acid were used at a level of 25 g/kg in a model cookie. Among these compounds, quercetin was found to be the most efficient in the inhibition of both glyoxal and fluorescent AGEs, while the impact on sensory properties was not evaluated [45]. Purified phenolics, namely, quercetin, gallic acid, ferulic acid and caffeic acid were also separately used in bread processing at levels in the range 1–20 g/kg. All phenolics tested were found to significantly reduce CML by 31.77%–87.56% even at the lowest concentration, with catechin being the most efficient. The sensory properties were not evaluated but it was hypothesized that the addition may adversely affect bread flavour, because it reduces the formation of pyrazines [44]. Alternatively, the whole dried pomace of red grape was added to a muffin recipe at 200 g/kg and was found to be effective in decreasing CML level with no significant changes in the sensory profile [30].

Another approach was to formulate grape phenolics in foods in order to recover high amounts of anti-glycation agent in the final product, which could potentially be absorbed and deliver antiglycation agents to the body. However, a significant loss of phenolics occurs during the cooking process. In fact, in bread with added grape phenolics, the loss of catechin, quercetin, gallic acid, ferulic acid and caffeic acid are 51%, 49%, 86%, 75% and 47%, respectively [44]. Hence, high levels of phenolics need to be incorporated to obtain significant residual amounts in the final product. Quercetin was added to bread at levels of 12–36 g/kg in order to develop a promising functional food with high antiglycation properties. However, by increasing the addition level of quercetin from 12 to 36 g/kg, there was a progressive negative impact on the steamed bread volume, which significantly decreased, as well as on the bread crumb texture, which increased in hardness. In contrast, incorporation levels below 1.20% had no impact. Moreover, within an enrichment in the range of 12 and 36 g/kg, quercetin negatively affected the yeast activity with significantly less CO_2_ produced in dough during proofing [46].

Alternatively, food matrices, such as tomato sauce and apple juice have very low AGEs content [15] indicating that they are not prone to AGE formation during processing, and hence can be used as vehicles for antiglycation agents.

A tomato puree with increased antiglycation activity was developed by adding micronized white grape skins at a level of 30 g/kg, achieving good liking scores by consumers. In this formulation, pasteurization slightly affected soluble proanthocyanidin content, for which recovery greater than 70% was achieved, while total flavonol content was not affected. In fact, quercetin and kaempferol glycosides and glucuronides decreased by about 30%, but the corresponding aglycones increased and the total amount remained constant, indicating that the main degradation was deglycosylation. As a result, the grape skin-fortified tomato puree had a 2.5-fold increase in the antiglycation activity in vitro [47]. A beverage based on apple puree formulated with 30 g/kg of micronized red grape skins was also developed, obtaining a good liking rate by consumers. Pasteurisation of apple puree beverage added with grape skins did not affect phenolic content. However, one-month storage in the temperature range 15–35 °C decreased the contents of anthocyanins, monomeric, dimeric and oligomeric flavanols, while the content of chlorogenic acid, flavonols and dihydrochalcones were unaffected. The antiglycation activity of the fortified apple puree was ~2-fold higher than the apple puree and was retained after one-month storage at 15 °C [48].

Alternatively, grape skin phenolics were encapsulated in alginate hydrogel as a controlled release matrix for antiglycation agents. The encapsulation efficiency was 68% and phenolics were almost completely retained at pH (gastric pH). Despite some phenolics interacting with alginate, which resulted in a decrease of their bioactivity, grape skinphenolics were almost completely released from the alginate microbeads at pH 7.4 (intestinal pH) and could exert antiglycation activity in vitro [49].

## 7. Conclusions

The antiglycation properties of grape skin phenolics have been proven by chemical studies elucidating their mechanism of action. Moreover, five randomized crossover trials have provided evidence that grape phenolics can prevent protein carbonylation in vivo and therefore can reach and protect target proteins. These studies open up a new challenge for healthy food formulation which is to develop grape phenolic-enriched foods in order either to prevent AGEs formation during processing and/or to deliver antiglycation compounds to the gut and human tissues. To advance knowledge already acquired on this topic, further investigations should better clarify the synergistic action among phenolic compounds in order to maximize their anti-AGE efficacy. Moreover, model systems should be designed to unravel the role of the food matrix on grape phenolic efficacy, ultimately leading to dietary strategies to prevent harmful consequences of AGEs formation.

## Figures and Tables

**Table 1:** In vivo studies on the inhibitory effects of grape phenolics on protein carbonylation.

Product and Daily Dose	Intake of Phenolics (Per Day)	Subjects	Trial Type	Duration	Outcome	Ref
Red grape skin extract powder (600 mg)	Total phenolic 31 mg	*n* = 15 Healthy	Crossover randomized	2 weeks	˃Glutathione reductase activity and glutathione peroxidase activity No effect on superoxide dismutase or catalase No effect on 2-aminoadipic semialdehyde, a plasma protein oxidation product No effect on malondialdehyde, a marker of lipoprotein oxidation.	[36]
Grape seed extract 2 tablets	Total phenolics 0.6 g	*n* = 32 Type-2 diabetic subjects	Crossover randomized, double-blind, placebo-controlled	4 weeks	<Fructosamine ˃High sensitivity C reactive protein ˃Reduced glutathione	[37]
Grape phenolics or grape phenolics + resveratrol 350 mg	~25 mg anthocyanins, ~1 mg flavonols, ~40 mg procyanidins, and ~0.8 mg hydroxycinnamic acids, or the same + 8.1 mg resveratrol	*n* = 75 Patients with stable coronary artery disease	Crossover randomized, triple-blind, placebo-controlled	1 year	˃Serum adiponectin <Plasminogen activator ˃Inhibitor type 1 <Atherothrombotic signals in peripheral blood mononuclear No effect on glycated haemoglobin	[38]
Grape polyphenols 6 capsules	Total phenolics 2 g	*n* = 38 Healthy overweight/obese first-degree relatives of type-2 diabetic subjects	Crossover randomized, double-blind, placebo-controlled	8 weeks	<Urinary F2-isoprostanes, muscle thiobarbituric acid reactive substances, muscle protein carbonylation ˃Hepatic insulin sensitivity	[39]
Red grape pomace (20 g)	Total phenolics 0.82 g	*n* = 38 Metabolic syndrome	Crossover randomized, placebo-controlled	16 weeks	<Carbonyl groups in plasma proteins (protein damage)	[40]
Pure compounds	Trans-resveratrol (90 mg) + hesperidine (120 mg)	*n* = 29 Overweight and obese subjects	Crossover randomized, double-blind, placebo-controlled	8 weeks	˃Expression and activity of glyoxalase-1 <Plasma methylglyoxal and total body methylglyoxal-protein glycation <Fasting and postprandial plasma glucose ˃Insulin sensitivity ˃Markers for vascular health	[41]
Pure compounds	Quercetin 3-glucoside (160 mg) (−)-epicatechin (100 mg)	*n* = 37 Healthy	Crossover randomized, double-blind, placebo-controlled	4 weeks	<Plasma methylglyoxal for quercetin 3-glucoside, no change in glyoxal, 3-deoxyglucosone and free and protein-bound AGE No effect for epicatechin	[42]

**Table 2:** Use of grape pomace-derived phenolics to inhibit AGE formation in foods or to increase the potential antiglycation activity of foods.

Food	Phenolic Source	Identified Phenolics	Outcome	Ref.
Wheat bread	Grape seed extract powder Integration: 2 g/kg bread	n.d.	CML level was 35 mg/kg in the bread crust of the control Addition of grape seed extract caused a 50% decrease in CML level	[43]
Wheat bread	Purified phenolics Integration: 1–20 g/kg flour	Flavanol: catechin Flavonol: quercetin Phenolic acids: gallic acid, ferulic acid, caffeic acids	CML level was 49.71 mg/kg in the bread crust of the control and 15.09 mg/kg in the bread crumb of the control Phenolics were found to significantly reduce CML (31.77%–87.56%)	[44]
Model cookie	Purified phenolics Integration: 25 g/kg	Flavonol: quercetin Flavanone: naringenin Flavanol: epicatechin Phenolic acids: rosmarinic acid, chlorogenic acid	Inhibition of fluorescent AGE formation was 80% for quercetin and ˂20% for naringenin, epicatechin, rosmarinic acid, chlorogenic acid	[45]
Muffin	Red grape pomace Integration: 200 g/kg	Phenolic acids: gallic acid Flavanols: catechin, epicatechin, oligomeric procyanidins Flavonols: quercetin 3-β-d-glucoside	CML level in the control muffins was 0.79–25.55 mg/kg depending on the receipt Red grape pomace decreased the level of CML up to 100%	[27]
Bread	Quercetin Integration: 12–36 g/kg	Flavonol: quercetin	Quercetin conferred antiglycation activity to bread	[46]
Tomato puree	White grape skin Integration: 30 g/kg	Flavonols: rutin, quercetin 3-*O*-glucuronide, quercetin 3-*O*-glucoside, quercetin, kaempferol 3-*O*-galactoside, kaempferol 3-*O*-glucuronide, kaempferol 3-*O*-glucoside, kaempferol Flavanone: naringenin Flavanols: oligomeric procyanidins	Addition of grape skins caused a 2.5-fold increase in the in vitro antiglycation activity of tomato (8 mmol catechin equivalents/kg in the fortified puree)	[47]
Apple puree	Red grape skin Integration: 30 g/kg	Anthocyanins: delphinidin 3-*O*-glucoside, cyanidin 3-*O*-glucoside, petunidin 3-*O*-glucoside, peonidin 3-*O*-glucoside, malvidin 3-*O*-glucoside Flavanols: catechin, epicatechin, procyanidin B2, procyanidin B1, oligomeric proanthocyanidins Phenolic acids: chlorogenic acid Dihydrochalcones: phloretin-2-*O*-xyloglucoside, phloridzin Flavonols: quercetin 3-*O*-glucoside, quercetin 3-*O*-glucuronide, quercetin, kaempferol	Addition of grape skins caused a 2.0-fold increase in the in vitro antiglycation activity of apple puree (69 mmol aminoguanidine equivalents/kg)	[48]
Alginate microcapsules	Red grape skin extract	Anthocyanins: delphinidin 3-*O*-glucoside, cyanidin 3-*O*-glucoside, petunidin 3-*O*-glucoside, peonidin 3-*O*-glucoside, malvidin 3-*O*-glucoside Flavanols: catechin, epicatechin, procyanidin B2, procyanidin B1 Flavonols: quercetin 3-*O*-glucoside, quercetin, kaempferol	The microbeads acted as pH-controlled release system for anti-glycation agents	[49]

n.d.: not determined; CML: Nε-(carboxymethyl) lysine.
